# Citrullinated histone H3 as a novel prognostic blood marker in patients with advanced cancer

**DOI:** 10.1371/journal.pone.0191231

**Published:** 2018-01-11

**Authors:** Charlotte Thålin, Staffan Lundström, Cedric Seignez, Maud Daleskog, Annika Lundström, Peter Henriksson, Thomas Helleday, Mia Phillipson, Håkan Wallén, Mélanie Demers

**Affiliations:** 1 Department of Clinical Sciences, Danderyd Hospital, Division of Internal Medicine, Karolinska Institutet, Stockholm, Sweden; 2 Palliative Care Services, Stockholms Sjukhem Foundation, Stockholm, Sweden; 3 Department of Oncology-Pathology, Karolinska Institutet, Stockholm, Sweden; 4 Department of Medical Cell Biology, Uppsala University, Uppsala, Sweden; 5 Department of Clinical Sciences, Danderyd Hospital, Division of Cardiovascular Medicine, Karolinska Institutet, Stockholm, Sweden; 6 Division of Translational Medicine and Chemical Biology, Department of Medical Biochemistry and Biophysics, Karolinska Institutet, Science for Life Laboratory, Stockholm, Sweden; Hospital for Sick Children, CANADA

## Abstract

Citrullinated histone H3 (H3Cit) is a central player in the neutrophil release of nuclear chromatin, known as neutrophil extracellular traps (NETs). NETs have been shown to elicit harmful effects on the host, and were recently proposed to promote tumor progression and spread. Here we report significant elevations of plasma H3Cit in patients with advanced cancer compared with age-matched healthy individuals. These elevations were specific to cancer patients as no increase was observed in severely ill and hospitalized patients with a higher non-malignant comorbidity. The analysis of neutrophils from cancer patients showed a higher proportion of neutrophils positive for intracellular H3Cit compared to severely ill patients. Moreover, the presence of plasma H3Cit in cancer patients strongly correlated with neutrophil activation markers neutrophil elastase (NE) and myeloperoxidase (MPO), and the inflammatory cytokines interleukin-6 and -8, known to induce NETosis. In addition, we show that high levels of circulating H3Cit strongly predicted poor clinical outcome in our cohort of cancer patients with a 2-fold increased risk for short-term mortality. Our results also corroborate the association of NE, interleukin-6 and -8 with poor clinical outcome. Taken together, our results are the first to unveil H3Cit as a potential diagnostic and prognostic blood marker associated with an exacerbated inflammatory response in patients with advanced cancer.

## Introduction

Cancer is a leading cause of morbidity and mortality [1], with considerable impact on society. Despite recent advances in oncology, diagnostics and prognostics remain major clinical challenges. In patients with advanced cancer, a correct determination of prognosis and monitoring of response to treatment permits more personalized therapy decision-making. Emerging interest is therefore being shed on possible diagnostic and prognostic blood markers.

Histones bind and package nuclear DNA into nucleosomes, which can be released into the blood stream upon cell activation or damage [2, 3]. As such, several clinical studies indicate that circulating cell free DNA (cfDNA) and nucleosomes serve as potential blood markers in a variety of malignancies [4–7], but investigations of the diagnostic or prognostic relevance of circulating histones are fewer, and focused mainly on sepsis and trauma patients [2, 8, 9]. Once released extracellularly, histones have been reported to mediate injurious effects on the host [2, 10–13], suggesting their potential as both prognostic markers and therapeutic targets.

Posttranslational histone modifications can have profound effects on their structure and function, and have been linked to various diseases [14]. Citrullinated histone H3 (H3Cit) is the product of the posttranslational conversion of peptidylarginine to citrulline on the N-terminal of histone H3. The subsequent decrease in positive charge of the histone residue results in a weaker binding to the negatively charged DNA, leading to chromatin decondensation [15]. Histone citrullination is catalyzed by the enzyme peptidylarginine deiminase 4 (PAD4) [16], primarily located in the cytoplasm of immune cells and the only PAD isozyme able to translocate to the nucleus upon cell activation [17]. This crucial role of H3Cit in immune cell chromatin decondensation has rendered H3Cit a central marker [15, 18, 19] of the recently described neutrophil release of decondensed and web-like nuclear chromatin, termed neutrophil extracellular traps (NETs). H3Cit can be detected in the nucleus of neutrophils upon stimulation [19] but also released into the bloodstream upon NETosis [20]. Furthermore, PAD4 has been shown to be overexpressed in various types of tumors [21], suggesting that H3Cit could be released into the bloodstream upon cancer cell death. Tumor cells may thus be an additional and unrecognized source of H3Cit in cancer.

First discovered to entrap and kill microorganisms as part of the innate immune system [22], the formation of NETs has later been defined as a novel cell death following strong stimulation [23]. Although neutrophil activation can induce the release of neutrophil granule proteins, such as neutrophil elastase (NE) and myeloperoxidase (MPO), into the blood stream without NET formation [24, 25], NE and MPO have been observed attached to the web-like NET-structures upon extrusion [22]. Recent data propose a central role of NETs in several non-infectious but inflammatory disease settings [26], including cancer. NETs have been shown to contribute to tumor progression [27–29], metastatic spread [30–33], and cancer-associated thrombosis [29, 34–36]. Tumor-released inflammatory cytokines have been shown to attract and prime neutrophils toward NETosis [34, 37]. H3Cit has been detected in murine [28, 38] and human tumors [27, 31, 32, 36], as well as in murine plasma [34] and a small number of cancer patients with thrombotic complications [36, 39].

Given emerging evidence on the central role of PAD4, histone citrullination, and NET formation in cancer, we sought to determine the levels of circulating H3Cit in a cohort of patients with advanced cancer. We report novel data on significant elevations of plasma H3Cit in cancer patients compared to both healthy individuals and severely ill and hospitalized patients without known cancer. Our results suggest a link between a cancer-associated inflammatory burden, neutrophil activation, and the release of H3Cit. Moreover, we provide a clinical significance of circulating H3Cit by showing associations between high levels of plasma H3Cit and an adverse clinical outcome.

## Materials and methods

### Patients and healthy controls

60 patients with a variety of advanced malignancies were included at the palliative in-patient unit at Stockholms Sjukhem, Stockholm, between October 2015 and March 2017. Inclusion criterion was active cancer, defined as diagnosis < 1 year and/or disseminated disease. Inclusion was conducted consecutively at all times when research personnel were available. There were no exclusion criteria. 50 healthy individuals were included as controls. Exclusion criteria were active or prior cancer diagnosis or the presence of comorbidities with the exception of hypertension. 51 severely ill and hospitalized patients without known active or prior cancer diagnosis were included at the Department of Medicine, Danderyd Hospital, as a second control group. Demographic data and comorbidity were obtained from medical records and patient history documented on admission. Comorbidity burden other than cancer was assessed by a weighed index taking into account the number and seriousness of comorbid disease by the use of Charlson Comorbidity Index (CCI) score [40], excluding score points for cancer. The study complied with the declaration of Helsinki, all patients and healthy individuals signed written informed consent. The protocol was approved by the Stockholm Ethical Review Board (dnr 2015/1533-31/1).

### Plasma analyses

Peripheral blood samples were drawn with the study participant in the supine position 30 min prior to blood sampling. Plasma samples were prepared from citrated whole blood following immediate centrifugation for 20 min at 2000 *g* at room temperature and stored at −80°C until further analyses. Plasma analyses were performed at the Clinical Research Center, Danderyd Hospital, and the Institute of Environmental Medicine, Karolinska Institute. At time of analyses, samples were thawed on ice once. Plasma H3Cit was quantified using a capture enzyme-linked immunosorbent assay (ELISA) as previously described [41]. Briefly, quantification of H3Cit in plasma samples was obtained using a biotinylated anti-histone antibody as capture antibody on streptavidin coated plate (Roche), and a rabbit anti-histone H3 citrulline antibody (Abcam) for detection. cfDNA, NE, MPO, and granulocyte colony-stimulating factor (G-CSF) were analysed with Quant-iT PicoGreen dsDNA assay (Invitrogen), PMN Elastase Human ELISA Kit (Abcam), Human Myeloperoxidase Quantikine ELISA kit (R&D Systems), and Human G-CSF Quantikine ELISA (R&D Systems) according to the instructions of the manufacturers. MPO-DNA complexes were identified using a capture ELISA as described previously [42]. Briefly, MPO-DNA complexes were captured on microplates pre-coated with monoclonal anti-MPO antibodies (Mercodia), and detected by monoclonal anti-DNA antibodies (Roche). IL-8, IL-6, TNFα, and IL-1β and were analysed with the MSD MULTISPOT Assay System (Meso Scale Diagnostics) in accordance with manufacturer’s instructions.

### Intracellular H3Cit

The portion of peripheral neutrophils positive for intracellular H3Cit was determined in a subset of 30 cancer patients, 17 hospitalized and severely ill patients without known cancer, and 27 healthy individuals. Citrated whole blood was drawn with the study participant in the supine position 30 min prior to blood sampling and centrifuged 10 min at 200 *g* at room temperature. White blood cell layer was collected and diluted in ammonium lysis buffer followed by centrifugation 5 min at 500 *g* at room temperature to separate and remove erythrocytes. Remaining white blood cells were washed in HBSS and centrifuged 5 min at 500 *g* at room temperature. After washing, cells were blocked with 10% Fetal Bovine Serum for 30 minutes on ice, washed twice in FACS Buffer, incubated with Fc-blocking antibody (BD Pharmingen) for 10 minutes on ice and then incubated with CD16-APC and CD66b-AF700 (Biolegend) for 30 minutes. After surface staining, cells were fixed and permeabilized with FoxP3 Staining Buffer Kit (eBioscience) according to manufacturer’s instructions and incubated for 30 minutes at room temperature with MMP9-FITC (RnDSystem) and H3Cit antibody (Abcam), washed twice in FACS Buffer before incubation with secondary anti-rabbit PE (RnDSystem) for 30 minutes. Cells were analysed on a Beckman Gallios flow cytometer (Beckman Coulter, Brea, CA, USA) with FlowJo software (Informer Technologies). Neutrophils were defined by using classic neutrophil surface markers CD16+ CD66b+ and the intracellular marker MMP9+. H3Cit expression in neutrophils was quantified and expressed as fold change to the mean of healthy donors.

### Statistical analyses

D'Agostino & Pearson normality test was used to test for normality of distribution, and statistical methods were chosen to fit non-normal distributions when appropriate. Categorical variables are presented as proportions and compared with the Fisher's exact test. Continuous variables are presented as medians with interquartile ranges (IQR) and compared with the Mann-Whitney U test. For the H3Cit ELISA, the limit of detection (LOD) was 10 ng/mL in samples diluted 1:2 [41]. Data was skewed to the right with a geometric standard deviation of 2.39. According to general praxis [43], we therefore replaced these values by LOD/√2, i.e. 7.1 ng/mL. Data were log transformed to obtain a normal distribution before Pearson correlation analysis. To verify the robustness of the correlations, we performed a multivariable Orthogonal Projection to Latent Structures (OPLS) regression to determine variables with a predictive influence on H3Cit levels, where a variable influence on projection (VIP) exceeding 0.8 was considered statistically significant [44]. Kaplan-Meier curves were constructed and survival analysis was performed using the log rank test obtaining hazard ratios (HR) between different levels of relevant variables and 100 days (short-term) mortality. OPLS statistics were analysed with SIMCA P+, version 14.1, (MKS Umetrics Ltd, Umeå, Sweden), and remaining statistical analyses were performed using GraphPad Prism 7 (GraphPad Software, Inc., La Jolla, CA, USA). A *P*-value < 0.05 was considered statistically significant.

## Results

### Study participants

In order to evaluate whether possible differences in circulating H3Cit reflected an underlying malignancy or merely a non-malignant disease burden, we included three groups of patients in our study; 60 patients with a variety of advanced malignancies, 51 hospitalized and severely ill patients without known cancer, and 50 healthy individuals (Table 1). There were no differences between the groups with regard to age or gender. However, severely ill patients without known cancer had a significantly higher non-malignant comorbidity burden than cancer patients (median comorbidity score 5.9 vs. 3.3, *P*<0.001), rendering this group suitable as a control group assessing the contribution of non-malignant disease burden. Demographic data, comorbidity, and tumor types are presented in Table 1.

**Table 1:** Demographic data, comorbidity and tumor types of study participants.

DEMOGRAPHIC DATA AND COMORBIDITY ALL STUDY PARTICIPANTS				
	Cancer patients (n = 60)	Severely ill patients without known cancer (n = 51)	p-value	Healthy (n = 50)
**Age, years (mean, SD)**	70.4 (12.4)	76.7 (11.5)	0.059	68.1 (7.7)
**Female (%)**	58	57	0.556	58
Comorbidity Index Score*	3.3 ± 1.8	5.9 ± 2.1	<0.001	0
**Comorbidity:**				
Hypertension—no. (%)	18 (30)	34 (67)	<0.001	13 (26)
Cerebrovascular disease—no. (%)	10 (17)	32 (63)	<0.001	0
Congestive heart disease- no. (%)	6 (10)	10 (20)	0.122	0
Renal insufficiency- no. (%)	7 (12)	9 (18)	0.266	0
Liver failure- no. (%)	3 (5)	11 (22)	0.009	0
Diabetes mellitus type 1- no. (%)	0 (0)	11 (22)	0.209	0
Diabetes mellitus type 2- no. (%)	9 (15)	13 (25)	0.127	0
Chronic pulmonary disease- no. (%)	4 (7)	9 (18)	0.067	0
Dementia- no. (%)	0 (0)	8 (16)	0.001	0
Acute infection- no. (%)	6 (10)	7 (14)	0.376	0
**TUMOR TYPES CANCER PATIENTS**				
**Adenocarcinomas:**	**No. (%)**	**Localized (no.)**	**Spread (no.)**	
Breast	10 (17)	0	10	
Colorectal	11 (18)	0	11	
Stomach	2 (3)	0	2	
Biliary tract	2 (3)	0	2	
Peritoneum	2 (3)	1	1	
Lung	6 (10)	1	5	
Prostate	5 (8)	0	5	
Gynecological	4 (7)	0	4	
Pancreas	2 (3)	0	2	
Unknown origin	2 (3)	0	2	
**Squamous cell carcinomas:**				
Esophagus	1 (2)	0	1	
Lung	1 (2)	0	1	
Gingival	1 (2)	0	1	
**Melanoma**	3 (5)	0	3	
**Glioblastoma**	3 (5)	1	2	
Other**	5 (8)	3	2	

SD, standard deviation

* Comorbidity Index Score calculated from the Charlson Comorbidity Index (CCI).

** Other primary tumor types were neuroendocrine (*N* = 1), sarcoma (*N* = 1), liposarcoma, (*N* = 1), lymphoma (*N* = 1) and acute myeloid leukemia (*N* = 1). Fisher's exact test for categorical data; Mann-Whitney *U* test for continuous data.

### Plasma levels of H3Cit are elevated in patients with advanced cancer

**Fig 1:**
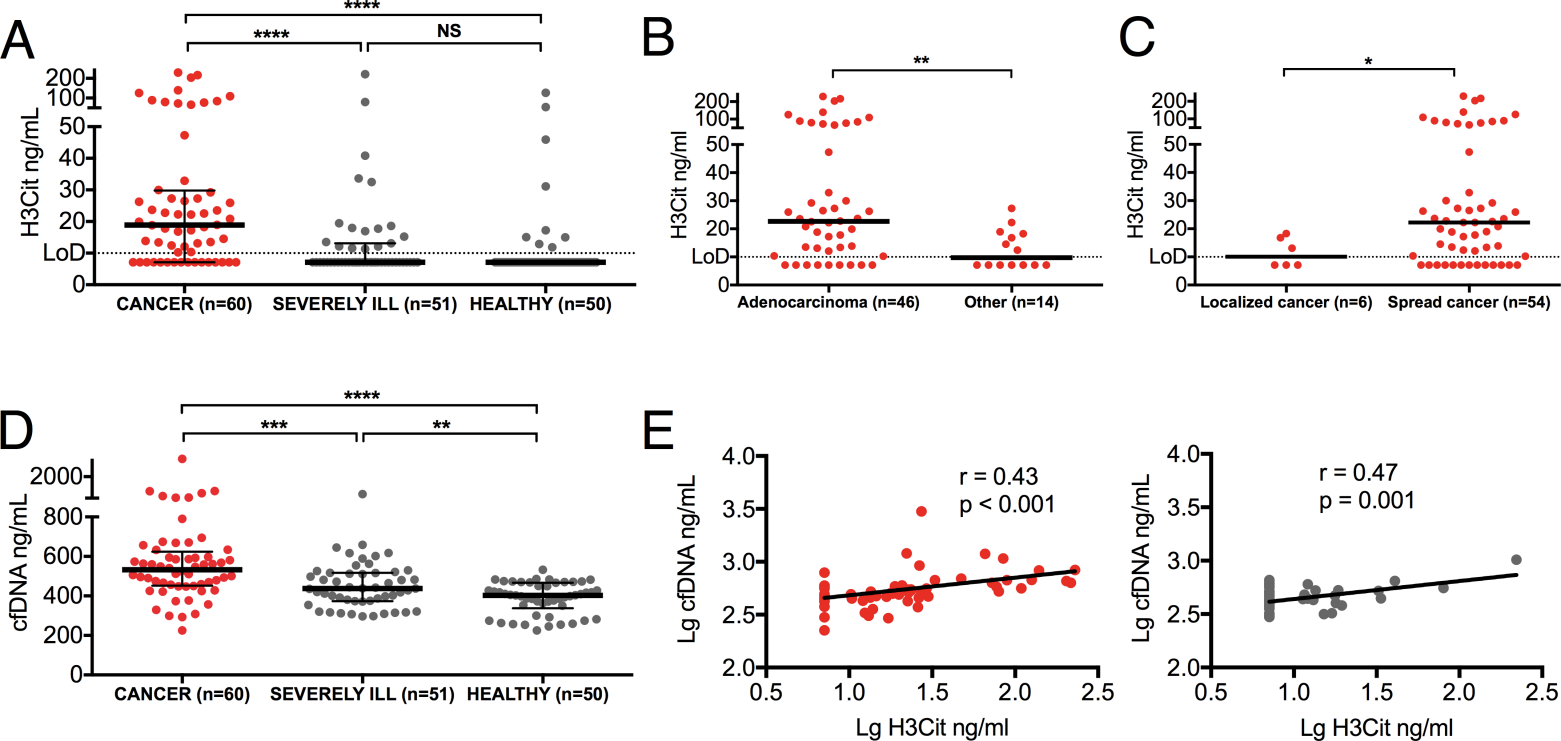
Elevations of plasma H3Cit and cfDNA in patients with advanced cancer. (*A*) Quantification of H3Cit showed increased levels in patients with advanced cancer compared to both healthy individuals and severely ill patients without known cancer. No significant difference in the levels of plasma H3Cit between severely ill patients without known cancer and healthy individuals were observed. (*B*) Plasma levels of H3Cit were significantly higher in patients with adenocarcinomas compared to patients with tumors of other histopathologies and *(C)* in patients with spread cancer compared to patients with localized tumors. *(D)* The levels of plasma cfDNA were significantly higher in cancer patients compared to both healthy individuals and severely ill patients without known cancer. The levels were also significantly higher in severely ill patients without known cancer compared to healthy individuals. (*E*) A significant positive correlation was found between plasma levels of H3Cit and cfDNA in cancer patients and in severely ill patients without known cancer. Lines represent medians with IQR. Groups were compared with the Mann-Whitney U test. Significance of correlation was analyzed with Pearson correlation coefficient after log transformed data to obtain a normal distribution. NS *P* > 0.05, * *P* < 0.05, ** *P* < 0.01, *** *P* < 0.001, **** *P* < 0.0001.

Quantification of plasma levels of H3Cit in our group of patients showed levels above the limit of detection in 73% of cancer patients, in 32% of severely ill patients without known cancer, and in 18% of healthy individuals. Cancer patients displayed a 3-fold increase in the median level of H3Cit compared to both healthy individuals (*P*<0.001), and severely ill patients without known cancer (*P*<0.001) (Fig 1A). Despite the high comorbidity burden in the severely ill patients without known cancer, these patients did not have significantly higher levels of H3Cit than those found in healthy individuals (*P* = 0.10), indicating that circulating H3Cit is not a reflection of general disease burden. Cancer patients presented with a variety of different tumor types and degree of metastatic spread (Table 1), and higher levels of H3Cit were observed in patients with adenocarcinoma (p = 0.005) and in patients with metastatic spread (p = 0.037) (Fig 1B and 1C).

Since histones are assumed to be released in complex with DNA, we proceeded to quantify the levels of cfDNA. Although there was a significant increase in cfDNA in cancer patients compared to severely ill patients without known cancer (*P*<0.001), the levels of cfDNA were also significantly higher in severely ill patients without known cancer compared to those found in healthy individuals (*P*<0.01), implying a non-malignant contribution to the release of circulating cfDNA (Fig 1D). As expected, the levels of H3Cit correlated positively with cfDNA in both cancer patients and severely ill patients without known cancer (Fig 1E). These results are the first to demonstrate an increase in circulating H3Cit in patients with advanced cancer. They also support that H3Cit is released in complex with cfDNA, but that circulating H3Cit, to a higher degree than cfDNA, reflects tumor burden, as opposed to a general and non-malignant disease burden.

### Plasma levels of H3Cit are associated with neutrophil activation in patients with advanced cancer

**Fig 2:**
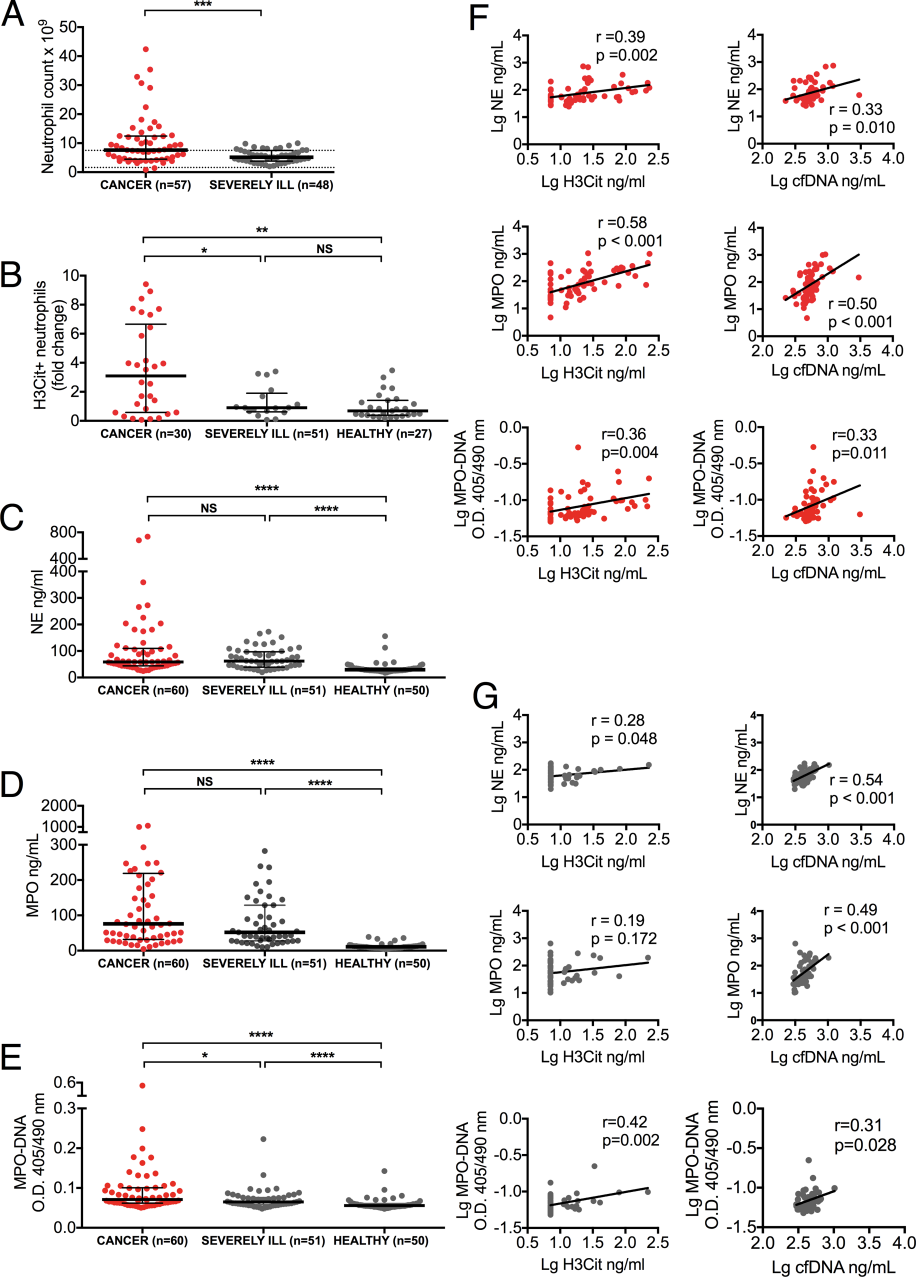
Cancer-associated neutrophil activation correlates with circulating H3Cit and cfDNA. (*A*) Peripheral neutrophil count was higher in cancer patients compared to severely ill patients without known cancer (dotted line represents upper reference interval). (*B*) Flow cytometry analysis showed a higher number of neutrophils positive for intracellular H3Cit in cancer patients compared to both healthy individuals and severely ill patients without known cancer. No significant difference was observed between severely ill patients without known cancer and healthy individuals. (*C-D*) Plasma NE (*C*) and MPO (*D*) were equally elevated in cancer patients and severely ill patients without known cancer compared to healthy individuals. *(E)* Plasma MPO-DNA complexes were significantly higher in cancer patients compared to both healthy individuals and severely ill patients without known cancer. The levels were also significantly higher in severely ill patients without known cancer compared to healthy individuals. *(F)* H3Cit and cfDNA correlated positively with NE, MPO and MPO-DNA complexes in cancer patients. (*G*) Similar correlations were seen in severely ill patients without known cancer, with the exception of correlations between H3Cit and NE and MPO. Lines represent medians with IQR. Groups were compared with the Mann-Whitney U test. Significance of correlation was analyzed with Pearson correlation coefficient after log transformed data to obtain a normal distribution. NS *P* > 0.05, * *P* < 0.05, ** *P* < 0.01, *** *P* < 0.001, **** *P* < 0.0001.

To assess whether the increase in circulating H3Cit seen in cancer patients reflected a systemic NET burden, we compared the number of peripheral neutrophils, portion of peripheral neutrophils positive for intracellular H3Cit, plasma markers of neutrophil activation, neutrophil elastase (NE) and myeloperoxidase (MPO) known to be released with NETs [22], and MPO-DNA complexes. Peripheral neutrophil count was significantly higher in cancer patients compared to severely ill patients without known cancer (*P*<0.001), with 18% of cancer patients displaying neutrophil counts exceeding twice the upper reference limit of 7.5 x 10^9^ cells/L, whereas none of the severely ill patients without known cancer displayed such neutrophilia (Fig 2A). Furthermore, there was a 3-fold increase in the portion of neutrophils positive for intracellular H3Cit in cancer patients compared to both healthy individuals (*P* = 0.002), and severely ill patients without known cancer (*P* = 0.02) (Fig 2B). As with circulating H3Cit, the portion of neutrophils positive for intracellular H3Cit did not differ between severely ill patients without known cancer and healthy individuals (*P* = 0.35). Plasma levels of NE and MPO were significantly elevated in cancer patients compared to healthy individuals (*P*<0.001), but they did not differ from the levels found in severely ill patients without known cancer (NE, p = 0.55; MPO, *P* = 0.08), indicating a similar degree of neutrophil activation in these two groups (Fig 2C and 2D). Plasma levels of MPO-DNA complexes were significantly increased in cancer patients compared to both severely ill patients without known cancer (p = 0.026) and healthy individuals (p<0.001), but they were also significantly higher in severely ill patients without known cancer compared to healthy individuals (p<0.001)(Fig 2E). Interestingly, cancer patients displayed positive correlations between plasma levels of H3Cit, cfDNA, markers of neutrophil activation, and MPO-DNA complexes (Fig 2F), suggesting that the presence of circulating H3Cit may be linked to neutrophil activation and NETs. Although severely ill patients without known cancer displayed similar correlations between cfDNA, markers of neutrophil activation, and MPO-DNA complexes, the correlations between H3Cit and markers of neutrophil activation were weaker or absent (Fig 2G), indicating a predominant neutrophil activation with minimal NET formation in these patients. The correlation between H3Cit and MPO-DNA complexes, however, remained significant in severely ill patients without known cancer. Taken together, these results suggest a similar level of neutrophil activation in patients with and without cancer, but a distinct state of neutrophil activation with an excess release of H3Cit, possibly through NETosis, in cancer patients.

### IL-8 and IL-6 are elevated in patients with advanced cancer and they correlate to the levels of H3Cit

**Fig 3:**
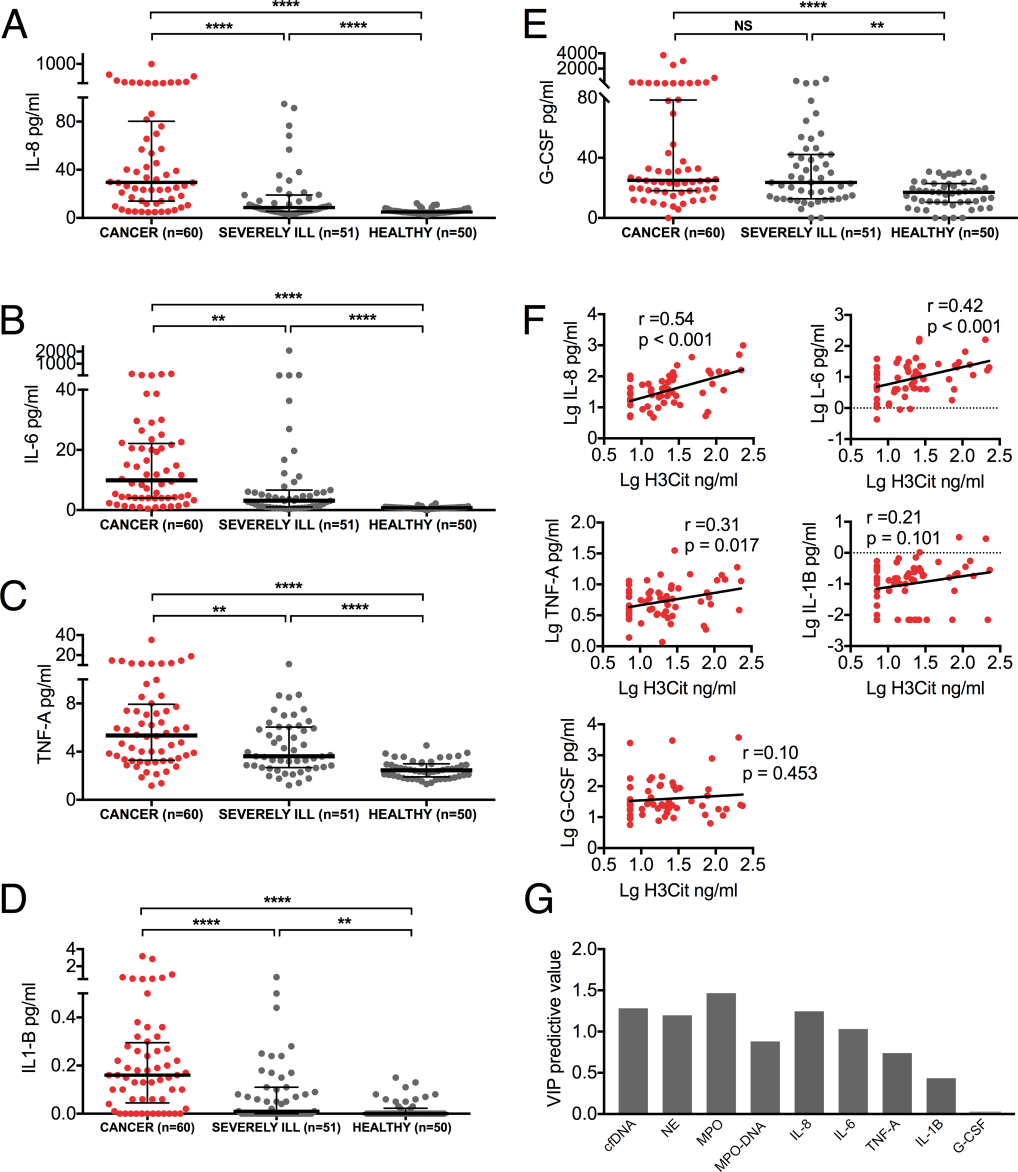
Inflammatory cytokines are elevated and correlate with H3Cit in patients with advanced cancer. (*A-E*) Plasma levels of IL-8, IL-6, TNFα, IL-1β, and G-CSF were all significantly higher in cancer patients compared to healthy individuals, as well as to severely ill patients without known cancer, with the exception of G-CSF which was similarly elevated in cancer patients and severely ill patients without known cancer. *(F)* Plasma levels of H3Cit correlated to plasma levels of IL-8 and IL-6, but weaker or no correlations were found to TNFα, IL-1β and G-CSF. (*G*) Multivariable regression confirmed the predictive influence of plasma cfDNA, NE, MPO, MPO-DNA complexes, IL-8, and IL-6 on plasma H3Cit levels (p<0.001). VIP, variable influence on projection. Lines represent medians with IQR. Groups were compared with the Mann-Whitney U test. Significance of correlation was analyzed with Pearson correlation coefficient after log transformed data to obtain a normal distribution. NS *P* > 0.05, * *P* < 0.05, ** *P* < 0.01, *** *P* < 0.001, **** *P* < 0.0001.

In search of a contributing factor to the distinct neutrophil activation leading to an excess NET formation in cancer patients, we evaluated the levels of inflammatory cytokines known to be elevated in cancer [45], and previously shown to induce NETosis [22, 34, 37, 46–49]. As expected, plasma levels of IL-8, IL-6, TNFα, IL-1β, and G-CSF were all significantly higher in cancer patients compared to healthy individuals (Fig 3A–3E). These levels were also, with the exception of G-CSF, significantly higher in cancer patients compared to those found in severely ill patients without known cancer. The levels of H3Cit in cancer patients correlated positively with IL-8 and IL-6, weakly with TNFα and no correlation was found with IL-1β and G-CSF (Fig 3F), suggesting a link between IL-8, IL-6, and the release of H3Cit. These correlations were weaker or absent in severely ill patients without known cancer (Table 2). To verify the robustness of the positive correlations found between H3Cit, neutrophil activation and inflammatory cytokines in cancer patients, we performed a multivariable OPLS regression to determine variables with a predictive influence on H3Cit levels. Consistent with Pearson correlations, we found that cfDNA, NE, MPO, MPO-DNA complexes, IL-8, and IL-6 had the strongest predictive influence on H3Cit (*P*<0.001) (Fig 3G). Taken together, these results support an exacerbated tumor-associated inflammatory burden with a distinct neutrophil activation contributing to the release of H3Cit in the blood stream of cancer patients.

**Table 2:** Correlations between plasma H3Cit and inflammatory cytokines in severely ill patients without known cancer.

	H3Cit	
	*R*	*P-*value
**IL-8**	0.37	0.007
**IL-6**	0.09	0.51
**TNF-A**	0.22	0.120
**IL-1B**	0.15	0.305
**G-CSF**	0.03	0.834

Significance of correlation were analyzed with Pearson correlation coefficient after log transformed data to obtain a normal distribution.

### High levels of plasma H3Cit are prognostic for short-term mortality in patients with advanced cancer

**Fig 4:**
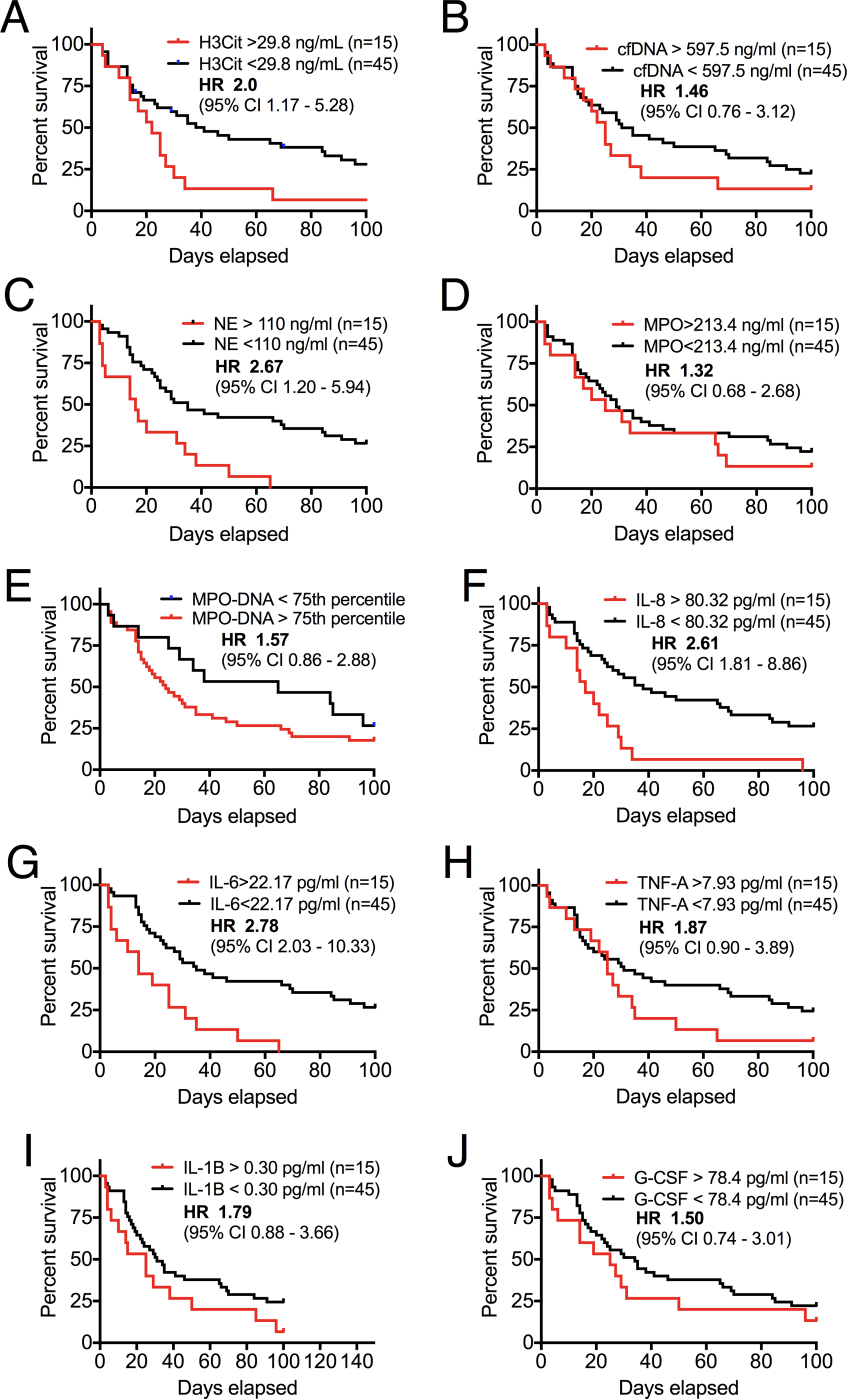
Prognostic value of high levels of plasma H3Cit, NE, IL-8 and IL-6 in patients with advanced cancer. Kaplan-Meier curves and survival analyses with the log rank test obtaining HR were performed to assess associations between laboratory markers and short-term (100 days) mortality. Patients with plasma H3Cit (*A*) levels above the 75^th^ percentile presented with a 2-fold increased risk for short-term mortality compared with patients with plasma levels below the 75^th^ percentile (*P* = 0.02). Increased levels of cfDNA (*B*) lacked prognostic significance (*P* = 0.24). Increased levels of NE (*C*) displayed a similar prognostic significance as high levels of plasma H3Cit (*P*<0.001). High levels of MPO (*D*) and MPO-DNA complexes *(E)* lacked prognostic significance (*P* = 0.39). The inflammatory cytokines IL-8 (*F*) and IL-6 (*G*) were also strongly associated with short-term mortality (*P* = 0.001 and <0.001 respectively). There was no significant association between high levels of TNFα (*H*), IL-1β (*I*) and G-CSF (*J*) and short-term mortality (*P* = 0.10, 0.12 and 0.27 respectively).

Considering the harmful effects of extracellular histones and excessive NET formation [10–12, 50, 51], along with prior data demonstrating a correlation between the presence of NETs and tumor burden [28, 32], we hypothesized that high levels of circulating H3Cit would predict poor outcome in cancer patients. Indeed, plasma H3Cit levels above the 75^th^ percentile (> 29.8 ng/mL) were associated with a 2-fold increased risk of short-term mortality (*P* = 0.02) (Fig 4A), whereas high levels of cfDNA lacked prognostic significance (*P* = 0.24) (Fig 4B). High plasma levels of NE displayed a similar prognostic value as H3Cit (*P*<0.001) (Fig 4C), which was not found with high levels of MPO (*P* = 0.39) or MPO-DNA complexes (*P* = 0.18) (Fig 4D and 4E). Interestingly, high levels of both plasma IL-8 and IL-6, the two cytokines with the strongest correlations to plasma H3Cit, were also associated with short-term mortality (*P* = 0.001 and <0.001 respectively) (Fig 4F and 4G), whereas high plasma levels of TNFα, IL-1β, and G-CSF lacked prognostic significance (*P* = 0.10, 0.12 and 0.27 respectively) (Fig 4H–4J). These results are the first to indicate a clinical and prognostic relevance of elevated levels of circulating H3Cit in cancer patients. They also support prior data on a prognostic significance of high levels of circulating NE [52], IL-8 [53] and IL-6 [54] in cancer, suggesting a link to cancer associated NETosis.

## Discussion

This is the first study to report elevations of circulating H3Cit in patients with advanced cancer not only in comparison to healthy individuals, but also in comparison to severely ill and hospitalized patients without known cancer. This elevation was found primarily in patients with adenocarcinoma and in patients with metastatic disease, types of cancer in which NETs have been described [30, 32, 34, 36, 51]. Despite high comorbidity burden in the group of severely ill patients without known cancer, these patients did not have higher levels of H3Cit than those found in healthy individuals, supporting that circulating H3Cit may be a marker for advanced cancer, and not merely for non-malignant disease burden. Moreover, we provide novel data on the potential prognostic significance of circulating H3Cit with a 2-fold increased risk for short-term mortality in cancer patients with high levels of plasma H3Cit.

Considering the critical role of histone citrullination in NET formation [15, 18, 19], as well as emerging reports on the central role of NETs in various malignancies [27, 28, 30, 31, 33–35, 51], neutrophil activation and NET formation seems to be a conceivable source of circulating H3Cit in cancer patients. In line with this, we found clear correlations between neutrophil activation, MPO-DNA complexes and plasma levels of H3Cit in cancer patients. Cancer patients also displayed an increase in the portion of peripheral neutrophils positive for intracellular H3Cit, assumed to be primed for NETosis. Surprisingly, plasma levels of NE and MPO did not differ between cancer patients and severely ill patients without known cancer, suggestive of a neutrophil activation without NET formation in the absence of malignancy. Although cfDNA, NE and MPO are all constituents of NETs, NE and MPO are also released upon neutrophil activation [25, 55], and a subsequent protease-induced tissue injury may contribute to the release of cfDNA [56]. Interestingly, although H3Cit was not increased in severely ill patients without known cancer compared to healthy individuals, MPO-DNA complexes were significantly elevated in these severely ill patients, and a positive correlation between H3Cit and MPO-DNA was observed. It is thus possible that plasma MPO-DNA complexes, in addition to being released in complex through NETosis, may be independently released into the circulation and subsequently form complexes through electrostatic interactions [57], questioning their specificity as NET markers.

The stimuli triggering NETosis in cancer are still unknown. Recently, Alfaro et al showed that neutrophils from healthy donors were stimulated toward NET formation if incubated in plasma from cancer patients, but not in plasma from healthy donors [37]. Thus, circulating tumor-derived and NET-inducing factors appear to be present in plasma from cancer patients. Our results suggest a new link between IL-8 and IL-6, neutrophil activation, and the release of H3Cit in the blood stream. IL-8 and IL-6 have been shown to be expressed and released by cancer cells [53, 58], and to induce NET formation in vitro [22, 37, 48]. The higher levels of these cytokines, and the high correlation to plasma H3Cit in cancer patients compared to severely ill patients without known cancer are supportive of a cancer-associated exacerbated inflammatory state, potentially inducing a distinct state of neutrophil activation, resulting in the priming of neutrophils toward NETosis and subsequent release of H3Cit.

Our results are the first to demonstrate a significant association between high levels of circulating H3Cit and short-term mortality. The present study does not reveal whether H3Cit contributes to the poor prognosis in these patients or if the histones merely reflect tumor burden and stage, but released in the bloodstream, histones have the potential to mediate detrimental effects on the host. Extracellular histones have been reported to contribute to endothelial and epithelial cell damage [11], thrombocytopenia [10], and thrombus formation [12]. As previously reported [52], high levels of NE also displayed a strong association with short-term mortality within the cancer group, although the levels were equally high in severely ill patients without known cancer, questioning a specificity for cancer. Dysregulated and excess release of NE may not only harm the host by degrading endothelial and epithelial tissue [59], but has also been shown to promote tumor cell proliferation [60]. These harmful effects may thus, in part, explain the poor clinical outcome in the cancer patients. Moreover, IL-8 and IL-6, the two cytokines with the strongest correlations to circulating H3Cit, were the only cytokines prognostic for short-term mortality in our cohort of cancer patients. Sanmamed et al recently showed that high levels of plasma IL-8 reflect tumor burden and treatment response in a variety of malignancies [53], and IL-6 has been proposed to have a prognostic value on survival in cancer patients [54], corroborating our results.

Even though H3Cit has a critical role in NET formation, and despite the above-discussed indications of neutrophil-released H3Cit in our cohort of cancer patients, we cannot rule out at least a partial tumor cell origin of the elevated levels of circulating H3Cit. As mentioned, PAD4, the histone-citrullinating enzyme, has also been found to be overexpressed in various tumors, whereas no PAD4 expression was found in benign tumors and non-cancerous inflamed tissue samples [21], suggesting abnormal PAD4 activity/citrullination in tumor cells. Moreover, Leshner et al previously showed that cancer cells overexpressing PAD4 were able to release decondensed chromatin in a process similar to that observed in NETosis [15]. Immunostaining revealed a strong presence of H3Cit on the extracellular web-like structures, as well as in cancer cells overexpressing PAD4. Considering the accumulating data on the role of PAD4 in various malignancies, cancer cells may thus be an unrecognized source of circulating H3Cit, with or without a concomitant systemic NET burden.

In conclusion, our results are the first to show an elevation, as well as a prognostic significance, of circulating H3Cit in cancer patients. We link the high levels of circulating H3Cit in cancer patients to an exacerbated inflammation and neutrophil activation, complementing emerging data on the central role of NETs in cancer. Our results also support previous data on the prognostic significance of high plasma levels of IL-8 and IL-6 in cancer patients, rendering a combination of these markers appealing in the quest for robust and specific prognostic markers in a cancer setting. It is, however, worth noted that both cancer patients and severely ill patients without known cancer comprise heterogeneous groups, and our findings are based on a limited number of patients preventing sub groups analyses within these groups. Furthermore, we were not able to perform extensive screening for occult cancer in the group of severely ill patients without known cancer, rendering a possible source of error. Larger studies are therefore warranted to assess whether circulating H3Cit, alone or in combination with other markers, could be implemented in diagnostics or prognostics in a clinical context of cancer.
